# 5α-Reductase Isoenzymes: From Neurosteroid Biosynthesis to Neuropsychiatric Outcomes

**DOI:** 10.3390/neurosci7010020

**Published:** 2026-02-02

**Authors:** Carmen Rodriguez-Cerdeira

**Affiliations:** 1 Fundación Vithas, Grupo Hospitalario Vithas, 28043 Madrid, Spain; carmencerdeira33@gmail.com; Tel.: +34-600538114; 2 Dermatology Department, Grupo Hospitalario (CMQ Concheiro), Manuel Olivié 11, 36203 Vigo, Spain; 3 Department of Health Sciences, University of Vigo, Campus of Vigo, As Lagoas, 36310 Vigo, Spain

**Keywords:** 5a-reductase, SRD5A1, SRD5A2, SRD5A3, neurosteroids, allopregnanolone, finasteride, dutasteride, depression, anxiety, suicidality

## Abstract

5a-reductase (5a-R) isozymes are essential for androgen metabolism and neurosteroid biosynthesis, linking endocrinology and neuropsychiatry. This systematic review, conducted in accordance with PRISMA 2020 guidelines, aimed to synthesize current evidence on the tissue distribution of SRD5A1, SRD5A2, and SRD5A3 and their implications in mental health. A systematic search of the PubMed, Scopus, and Web of Science databases up to February 2025 identified 257 articles, of which 83 met the inclusion criteria. SRD5A1 is broadly expressed in the liver, skin, and central nervous system, contributing to allopregnanolone synthesis; SRD5A2 is mainly restricted to androgen-dependent tissues, playing a key role in prostate development and alopecia; and SRD5A3 is associated with glycosylation processes and oncogenesis. Converging evidence suggests that impaired neurosteroidogenesis due to 5α-R inhibition may underlie vulnerability to anxiety, depression, and suicidality. While earlier epidemiological findings were heterogeneous, recent pharmacovigilance data have strengthened the evidence supporting this association. Pharmacovigilance and clinical reports show that a subset of patients treated with finasteride or dutasteride may experience persistent psychiatric and sexual adverse effects, known as post-finasteride syndrome. The current findings underscore the need for careful patient counseling, systematic monitoring, and further translational studies integrating genetics, neuroendocrine markers, and standardized psychiatric outcomes to identify individuals at risk and advance personalized medicine in this field.

## 1. Introduction

The steroid 5α-reductase (5α-R) enzyme family catalyzes the irreversible conversion of testosterone into dihydrotestosterone (DHT), a potent androgen that plays a key role in male sexual differentiation, prostate development, and hair follicle regulation. Beyond its classical role in androgen metabolism, 5α-reductase also contributes to the synthesis of neurosteroids such as allopregnanolone from progesterone, thereby influencing mood, cognition, and stress response. Han et al. [1] characterized the crystal structure of steroid 5-alpha-reductase (SRD5A), confirming its conserved nicotinamide adenine dinucleotide phosphate (NADPH)-dependent mechanism for steroid reduction and underscoring its role as a cornerstone in endocrine and neuroendocrine physiology. Radmayr et al. [2] described the essential function of this enzyme in the conversion of testosterone into dihydrotestosterone (DHT), a key androgen in human development. Traish [3] expanded this view, emphasizing that beyond its classical role in reproductive biology, 5α-reductase also contributes to neurosteroid biosynthesis and the regulation of emotional and behavioral processes. Sánchez et al. [4] pointed out that the type I isoenzyme is abundant in peripheral tissues and in the brain, where it initiates the synthesis of allopregnanolone from progesterone. Azzouni et al. [5] reported that type II isoenzyme predominates in androgen-dependent organs, playing a pivotal role in male sexual development and urological diseases. More recently, Cantagrel et al. [6] identified type III 5α-reductase as being involved in glycosylation and prostate carcinogenesis, although its role in the central nervous system (CNS) remains unclear. Finally, Melcangi et al. [7] emphasized that this dual distribution highlights the enzyme’s relevance both as a peripheral androgenic enzyme and as a regulator of neuroendocrine function within the CNS.

The distribution of 5α-reductase isoenzymes is not limited to peripheral androgenic tissues but also extends to the brain, where they initiate the synthesis of neurosteroids. Schiller et al. [8] demonstrated that allopregnanolone is synthesized from progesterone through the sequential actions of 5α-reductase and 3α-hydroxysteroid dehydrogenase. Joshi et al. [9,10] showed that this neurosteroid acts as a potent positive allosteric modulator of the gamma-aminobutyric acid (GABA)-A receptor, enhancing GABAergic neurotransmission and contributing to anxiolytic, sedative, anticonvulsant, and mood-stabilizing effects. Liang et al. [11] showed that 5α-reductase activity therefore contributes indirectly to the regulation of emotional and behavioral homeostasis, extending its role beyond the classical androgen axis.

The discovery of specific 5α-reductase inhibitors—primarily finasteride (selective for type II isoenzyme) and dutasteride (active against types I and II)—represented a major therapeutic advance in urology and dermatology. These agents have become standard treatments for benign prostatic hyperplasia (BPH) and androgenetic alopecia (AGA), with an overall safety profile considered favorable [12,13]. However, since the early 21st century, it has been observed that some patients treated with 5α-reductase inhibitors report neuropsychiatric symptoms such as anxiety, depression, and cognitive dysfunction. Moreover, a persistent condition following treatment discontinuation, termed post-finasteride syndrome (PFS), has been described, encompassing both somatic and psychological manifestations [14,15]. The description of PFS has generated intense debate. While the absence of universally accepted diagnostic criteria has contributed to controversy, it is important to note that this controversy may have been amplified by industry strategies, as described by Michaels in Doubt is Their Product Nevertheless, the convergence of patient reports and mechanistic plausibility has been sufficient for international regulatory agencies—namely the Medicines and Healthcare products Regulatory Agency (MHRA, United Kingdom), the European Medicines Agency (EMA, European Union), and the U.S. Food and Drug Administration (FDA)—to mandate explicit warnings in the labeling of these drugs [16]. In parallel, several epidemiological and pharmacovigilance studies have attempted to clarify the relationship between 5α-reductase inhibitor use and the risk of suicidal ideation [17]. Melcangi et al. [18] reported altered neuroactive steroid levels associated with psychiatric and andrological symptoms in post-finasteride patients [18]. Recent pharmacovigilance analyses have identified consistent disproportionality signals linking finasteride to suicidality [19]. These signals indicate a reporting association but do not establish causality.

From a clinical perspective, however, the strength of the evidence supporting finasteride use is more limited than often assumed [14,15,17,19]. At the genetic level, emerging evidence suggests that individual susceptibility to post-finasteride syndrome may be influenced by specific polymorphisms [18,20]. A recent case series identified genetic variants potentially associated with neuropsychiatric vulnerability in patients affected by this condition, highlighting the need to consider genetic predisposition when evaluating the safety profile of 5α-reductase inhibitors [20]. Furthermore, it should be noted that reference [12] refers to topical finasteride, for which the FDA has recently issued a safety warning.

The objective of this review is to provide an integrative, evidence-based perspective on the neuropsychiatric aspects of 5α-reductase, with particular focus on (1) its relationship with allopregnanolone synthesis and mood modulation; (2) the quality and limitations of the clinical evidence, including reports of anxiety, depression, and suicidal ideation associated with enzyme inhibitors; and (3) the possible existence of genetic predisposition accounting for individual vulnerability.

A summary of the main findings regarding isoenzymes, neurosteroid synthesis, psychiatric manifestations, clinical studies, and genetic evidence is presented in Table 1.

## 2. Materials and Methods

A systematic review of the scientific literature on the enzyme 5α-R and its neuropsychiatric implications was conducted, with emphasis on the relationship between neurosteroid synthesis (particularly allopregnanolone) and mood regulation. The search was performed in the PubMed/MEDLINE, Scopus, and Web of Science databases and Google Scholar databases, including articles published up to November 2025.

Data regarding identification, selection, inclusion, and exclusion of studies were collected and organized according to the PRISMA flow diagram to ensure transparency and traceability throughout the review process.

### 2.1. Search Terms

Combinations of keywords and MeSH descriptors in English and Spanish were used:

- “5-alpha reductase” OR “SRD5A”.
- “neurosteroids” OR “allopregnanolone”.
- “mood disorders” OR “depression” OR “anxiety” OR “suicidal ideation”.
- “finasteride” OR “dutasteride” OR “5-alpha reductase inhibitors”.
- “genetic polymorphism” OR “susceptibility”.

The search strategy combined these terms with the Boolean operators AND and OR to maximize sensitivity.

### 2.2. Inclusion Criteria

- Original articles, systematic reviews, meta-analyses, and case series published in English or Spanish.
- Human and animal studies addressing the following:
  1. Isoenzymes and distribution of 5α-reductase.
  2. The role of 5α-R in neurosteroid synthesis.
  3. Association of 5α-R inhibitors (finasteride, dutasteride) with neuropsychiatric symptoms.
  4. Genetic evidence of predisposition to adverse effects.

### 2.3. Exclusion Criteria

- Articles not available in full text.
- Publications with poor methodological quality (assessed based on study design, sample size, and validity of conclusions).
- Gray literature (conference abstracts, unpublished theses, non-peer-reviewed communications).

### 2.4. Study Selection and Analysis

**Figure 1:**
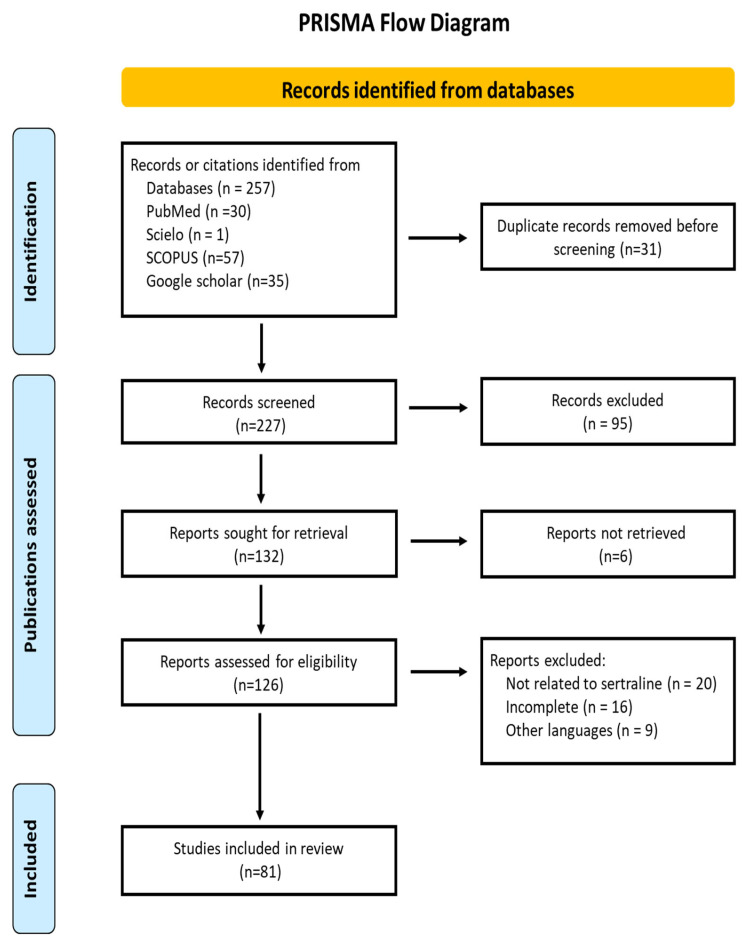
PRISMA flow diagram for the systematic review of the antifungal properties of sertraline, including database and register searches. The number of records from each source is specified. No automation tools were used in the screening process.

The systematic search identified a total of 257 records from PubMed, Scopus, and Web of Science. After the removal of 48 duplicates, 209 unique articles were screened by title and abstract. Of these, 128 were excluded for not meeting the inclusion criteria (e.g., unrelated topic, inadequate methodological quality, or language restrictions). The full text of the remaining 81 studies was reviewed in detail and included in the qualitative synthesis. No automation tools were used in the screening process. The study selection and data extraction were performed independently by two reviewers. The entire process followed the PRISMA 2020 guidelines [33] and is summarized in the flow diagram (Figure 1). Although registration in PROSPERO was not performed, the methodology adheres to PRISMA-P standards to ensure transparency, minimize bias, and avoid duplication.

### 2.5. PRISMA Statement

This review was conducted in accordance with the PRISMA 2020 guidelines for reporting systematic reviews [33]. The protocol was not registered in PROSPERO or any other database.

## 3. Main Findings of Literature Review

The following sections summarize the main findings of the reviewed literature, organized according to the distribution and functions of 5α-reductase isoenzymes, their role in neurosteroid synthesis, the psychiatric manifestations associated with their inhibition, and the clinical and translational evidence available to date. This structure allows for a comprehensive overview while highlighting both mechanistic insights and clinical implications. The main evidence is summarized in Table 1.

The reviewed studies confirm the existence of three main isoforms of 5α-reductase, namely, SRD5A1, SRD5A2, and SRD5A3, each encoded by distinct genes and characterized by differential tissue distribution. The type I isoenzyme (SRD5A1) is widely expressed in the liver, skin, and, most notably, CNS, including the cerebral cortex and cerebellum. Its enzymatic activity enables the conversion of progesterone into dihydroprogesterone, the initial step in allopregnanolone synthesis [34].

The type II isoenzyme (SRD5A2), localized primarily in androgen-dependent tissues such as the prostate and seminal vesicles, plays a pivotal role in male sexual development and in urological disorders, including BPH and AGA [35].

The type III isoenzyme (SRD5A3), more recently described, has been associated with glycosylation processes and prostate carcinogenesis. Although its function in the CNS has not been clearly established, a potential role in cerebral homeostatic mechanisms cannot be excluded [36].

More recently, Diviccaro et al. [21] confirmed the enzymatic steps leading to allopregnanolone synthesis, while Maguire et al. [22] reviewed its mechanistic role and clinical implications. Clinical studies reinforce this link: Fedotcheva et al. [23] reported decreased levels of allopregnanolone in patients with major depression, anxiety, and epilepsy. Collectively, these findings support the central role of 5α-reductase in neurosteroid synthesis and its relevance for affective disorders.

A marked decline in this neurosteroid has also been documented during the postpartum period, a circumstance that correlates with the risk of postpartum depression [24].

The growing interest in the 5α-R–allopregnanolone pathway has led to the development of therapies based on analogs of this neurosteroid. Clinical trials with brexanolone [25], the first formulation of allopregnanolone approved for clinical use, demonstrated a rapid and significant reduction in symptoms of postpartum depression, with remission rates exceeding 50% in some studies [26]. Similarly, zuranolone, an oral positive modulator of the GABA-A receptor inspired by allopregnanolone, has shown efficacy both in postpartum depression and in episodes of treatment-resistant major depressive disorder [27]. These translational findings underscore the importance of the metabolic pathway initiated by 5α-R in mood physiology. Unlike conventional antidepressants, which typically require several weeks to demonstrate efficacy, allopregnanolone analogs act within a matter of days, representing a significant therapeutic advance [27].

The use of 5α-R inhibitors—primarily finasteride (selective for SRD5A2) and dutasteride (active against SRD5A1 and SRD5A2)—is classically associated with clinical benefits in BPH and AGA. However, since the early 2000s, neuropsychiatric symptoms have been reported in a subset of patients treated with these drugs. Among the most frequently described are anxiety, dysphoria, memory and concentration disturbances, and depressive episodes [37].

In some cases, these symptoms persist even after discontinuation of treatment, leading to the description of PFS. This condition includes persistent sexual dysfunction, anhedonia, insomnia, suicidal ideation, and cognitive impairment, along with biochemical findings of reduced progesterone-derived neurosteroids [38,39].

Both the MHR [40] and the EMA [41] have issued specific warnings in the labeling of these drugs, alerting clinicians and patients to the potential emergence of depressive symptoms and suicidal ideation. The official recognition of these adverse effects has prompted strengthened pharmacovigilance measures and greater regulatory attention to the long-term safety of 5α-reductase inhibitors.

The association between 5α-reductase inhibitors and suicide risk has been explored in several epidemiological and pharmacovigilance studies. Among men treated for BPH, findings are heterogeneous, with some studies reporting an increased risk of suicidal ideation or behavior and others showing no clear association. In contrast, postmarketing analyses in androgenetic alopecia have more consistently identified signals of depression, anxiety, and suicidality. An overview of the principal studies, including study design, populations, and key outcomes, is provided in Table 2.

Discrepancies can be explained by confounding factors such as age, therapeutic indication, and the presence of pre-existing psychiatric comorbidity. Regulatory authorities have officially acknowledged that finasteride can cause depressed mood, depression, or suicidal thoughts. The European Medicines Agency required updates to product information and the inclusion of a patient card for 1 mg tablets used in androgenetic alopecia, explicitly stating, “Finasteride tablets can cause depressed mood, depression or suicidal thoughts” [41].

Current evidence indicates that not all patients exposed to 5α-R inhibitors develop neuropsychiatric effects. This has led to the exploration of genetic susceptibility hypotheses. Preliminary studies have identified polymorphisms in the SRD5A2 gene (e.g., the V89L variant), which may be associated with differences in enzymatic activity and, consequently, with variable vulnerability to depressive symptoms during treatment [6].

In addition, variations in GABA-A receptor subunits (such as GABRG2) have been described, which may modulate the response to altered allopregnanolone levels [31].

Other factors—including psychiatric history, psychosocial stress, and epigenetic variations—also appear to significantly influence individual risk. Although the available data derive from small samples and predominantly Asian populations, these studies reinforce the possibility of developing future genetic and epigenetic biomarkers to predict susceptibility to neuropsychiatric adverse effects associated with 5α-R inhibition [32,42]. To contextualize these findings, Table 1 summarizes the key evidence on isoenzymes, neurosteroid synthesis, psychiatric effects, and clinical translation.

Collectively, these data highlight the potential contribution of genetic variability in neurosteroid-related pathways to individual differences in susceptibility to neuropsychiatric effects associated with 5α-reductase inhibition (Table 3).

## 4. Discussion

The relationship between 5α-reductase inhibition and neuropsychiatric symptoms has evolved from a marginal concern to a central topic in contemporary scientific debate. Accumulating evidence indicates that finasteride, and to a lesser extent dutasteride, can induce neuropsychiatric adverse reactions, including anxiety, dysphoria, anhedonia, insomnia, cognitive complaints, and suicidal ideation [30].

Importantly, the available evidence fulfills several Bradford–Hill criteria for causality, including temporality, reversibility after drug discontinuation and recurrence upon rechallenge, consistency and replicability of findings across studies conducted in different countries and using diverse research designs, strong statistical significance, and robust biological plausibility. In line with these data, the European Medicines Agency has formally acknowledged that finasteride may cause depressed mood, depression, and suicidal thoughts, underscoring the clinical relevance of these neuropsychiatric adverse effects [41].

From a mechanistic perspective, 5α-reductase catalyzes the conversion of progesterone and deoxycorticosterone into neurosteroid precursors such as allopregnanolone, a positive allosteric modulator of the GABA-A receptor [43,44,45]. Allopregnanolone enhances GABA-dependent chloride currents and reduces neuronal excitability, exerting anxiolytic, antidepressant, and anticonvulsant effects [44,46]. Inhibition of 5α-reductase may therefore reduce neurosteroid synthesis and diminish GABAergic tone, predisposing susceptible individuals to dysphoria, anxiety, sleep disturbances, and affective symptoms [18,43]. This hypothesis is consistent with the demonstrated efficacy of allopregnanolone analogs (e.g., brexanolone and zuranolone) in postpartum depression and major depressive disorder [25,47].

Neurosteroids also modulate serotonergic, noradrenergic, dopaminergic, and glutamatergic systems, amplifying the integration of stress responses and resilience mechanisms [46,48]. Their reduction can lower the threshold for emotional decompensation, depending on genetic load, trauma history, comorbidity, and environmental stressors. Allopregnanolone further influences the amygdala, hippocampus, and medial prefrontal cortex, regions crucial for threat detection, emotional memory, and executive control [49,50]. Dysregulation at this level may disrupt synchrony between the default mode and executive networks, fostering attentional biases toward negative stimuli and rumination—well-documented mechanisms in depression and anxiety [51]. Additionally, reduced neurosteroid tone impacts the mesocorticolimbic dopamine system and serotonergic circuits, potentially explaining anhedonia and apathy reported by some patients during 5α-R inhibitor treatment [52].

Animal studies support this framework: experimental inhibition or genetic manipulation of 5α-R induces anxiety-like behaviors and cognitive deficits, aligning with clinical observations in susceptible subgroups [53,54].

The clinical literature, however, remains heterogeneous. In older men with BPH, population-based cohorts and pharmacovigilance databases suggest a modestly increased risk of depression, self-harm, or suicidal ideation [28,29,55,56]. By contrast, in younger men with AGA, available postmarketing pharmacovigilance data have consistently revealed disproportionality signals of depression, anxiety, and suicidality [19].

These discrepancies likely reflect confounding factors (age, comorbidity, polypharmacy, baseline depression), indication-specific psychosocial impact (AGA itself may trigger emotional burden), and differences in study design. To synthesize this evidence, the main epidemiological studies are summarized in Table 2.

Beyond these cohort findings, case series and observational reports have described clusters of persistent symptoms after discontinuation of finasteride, termed PFS [55,57,58]. Although the absence of standardized diagnostic criteria and the potential for reporting bias complicate its quantification, the consistency of reports and mechanistic plausibility support the need for precaution and structured follow-up.

**Figure 2:**
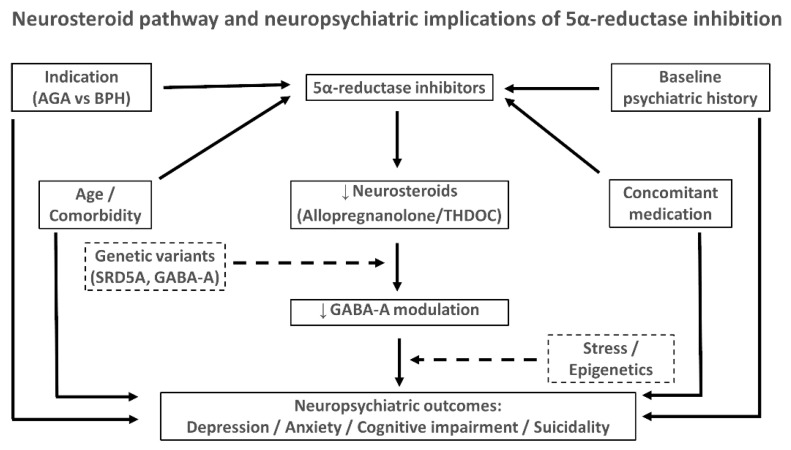
Inhibitors of 5α-reductase reduce neurosteroid synthesis, thereby decreasing GABA-A receptor modulation and potentially contributing to depression, anxiety, cognitive impairment, and suicidality. Confounders (psychiatric history, indication, age/comorbidity, concomitant medications) and effect modifiers (genetic variants, stress, epigenetic factors) influence individual vulnerability. Arrows: black = causal; gray = confounding; dashed = effect modification. Abbreviations: AGA, androgenetic alopecia; BPH, benign prostatic hyperplasia; SRD5A, steroid 5α-reductase; GABA-A, gamma-aminobutyric acid type A receptor; THDOC, tetrahydrodeoxycorticosterone.

Not all exposed individuals develop symptoms, highlighting the role of individual susceptibility. Polymorphisms in SRD5A2 (e.g., V89L) and variants in GABA-A receptor subunits have been proposed as biological moderators [32,59]. In addition, adverse life events, trauma, occupational stress, sleep deprivation, psychiatric comorbidity, and substance use may contribute to vulnerability, interacting with the pharmacological effect of 5α-R inhibitors [60]. This “vulnerability–resilience” framework explains why some patients tolerate treatment without difficulty while others experience marked neuropsychiatric effects [61]. This integrative framework is summarized in Figure 2**,** which illustrates the interaction of 5α-reductase inhibition, neurosteroid deficits, genetic variants, psychosocial stressors, and clinical modifiers in shaping neuropsychiatric outcomes. A synthesis of the candidate genes and variants is presented in Table 3**.**

When addressing methodological considerations and sources of bias, it is essential to acknowledge recurrent limitations in the available evidence. First [62], most studies are non-randomized observational designs, vulnerable to confounding by indication and to unmeasured variables (e.g., severity of BPH/AGA, social support, life events). Second [62], exposure is often inferred from dispensed prescriptions, which do not guarantee actual adherence or effective dosing. Third [63], psychiatric outcomes are frequently identified through administrative codes, with imperfect sensitivity and specificity, leading to underestimation of subclinical symptoms relevant to patients. Fourth [64], healthy survivor bias may occur if individuals experiencing adverse effects discontinue treatment early and thus “disappear” from databases. Fifth [62], temporal confounding adds noise: treatment initiation and emotional events (job changes, bereavement, illness) may coincide by chance. Finally, the literature is subject to publication bias: series with striking findings are more likely to be published, whereas negative studies remain unpublished. These limitations call for caution in drawing strong conclusions and highlight the need to prioritize prospective designs with standardized psychiatric assessments and direct measurement of neurosteroid levels.

Clinical practice requires careful consideration of the magnitude of therapeutic benefits against the possibility, albeit infrequent, of neuropsychiatric adverse events. In urology, administration of 5α-R inhibitors (finasteride, dutasteride) has been shown to reduce prostate volume, improve lower urinary tract symptoms, and decrease the risk of acute urinary retention and the need for surgery [65].

In dermatology, finasteride and dutasteride are effective options for stabilizing AGA and promoting partial hair regrowth [66,67].

The following paragraph should be rewritten: “On this basis, a pragmatic approach with reinforced safety measures can be proposed: (1) explicit pre-treatment information, clearly communicating the potential for neuropsychiatric effects in certain subgroups [68]; (2) brief baseline screening focused on mood, anhedonia, sleep quality, history of depression, anxiety or PTSD, substance use, and concomitant psychotropic medications [69]; (3) careful candidate selection, avoiding initiation in patients with uncontrolled depressive episodes or active suicidal ideation [70]; (4) dosing and follow-up based on the lowest effective dose, with reassessment at 4–8 weeks and subsequently at three-month intervals during the first year, including simple patient-reported scales of mood, anxiety, sleep, libido, and cognition [71]; (5) prompt management of emerging symptoms, including dose reduction, discontinuation, or psychiatric referral if necessary [72]; and (6) explicit documentation of informed consent, particularly in AGA, where the cosmetic nature of the indication lowers the tolerance for adverse effects [68]. This conservative and pragmatic approach maximizes patient safety without restricting access to the clinical benefits of 5α-R inhibitors.”

Several avenues deserve further development. Biomarkers such as plasma or CSF neurosteroid quantification, fMRI connectivity signatures, and MRS GABA measures may allow risk stratification [73]. Genetics and epigenetics could provide predictive panels of SRD5A1/2 and GABA-A variants, along with methylation markers of stress regulation [74]. Pragmatic trials with standardized psychiatric assessments, adaptive Bayesian designs, and N-of-1 approaches could improve causal inference [75,76,77]. Preclinical studies with isoenzyme-selective inhibitors and strategies that preserve CNS neurosteroid synthesis while acting on peripheral tissues may minimize central risks [78]. Strengthened pharmacovigilance platforms capable of detecting subclinical symptoms and monitoring dose/response relationships are also essential. Zhong et al. [79] provided a multidimensional pharmacovigilance assessment of finasteride, identifying diverse adverse events and reinforcing the need for cautious clinical monitoring.

Taken together, the evidence indicates that 5α-R inhibitors do not affect all users equally; consistent neuropsychiatric adverse effects have been documented in men treated for androgenetic alopecia, suggesting a higher susceptibility in this population [43,80]. Biological plausibility is well supported by the physiology of neurosteroids and their effects on GABA-A and affective networks, but the magnitude and distribution of risk depend on individual factors and the methodological biases inherent to observational evidence [81]. Therefore, a prudent clinical approach—clear patient information, baseline screening, use of the lowest effective dose, systematic follow-up, and coordination with mental health services when appropriate—offers a balanced path between therapeutic benefit and safety. The priority is to identify who should be treated, with which dose, and for how long, in order to maximize urological and dermatological benefits without compromising the patient’s mental health [19].

A summary of the main findings on 5α-reductase isoenzymes, neurosteroid synthesis, psychiatric manifestations, and translational evidence is provided in Table 1 [82], and the main genetic susceptibility factors are detailed in Table 3.

A comprehensive clinical approach is warranted in patients treated for androgenetic alopecia (AGA). Although AGA is not a psychiatric disorder per se, it is frequently associated with body image concerns, reduced self-esteem, and emotional distress. A recent systematic review and meta-analysis has confirmed the significant psychosocial impact of AGA, particularly in men. In this context, non-pharmacological interventions such as cognitive-behavioral therapy, mindfulness-based stress reduction, supportive counseling, and structured coping strategies may be beneficial in addressing psychological distress [83].

## 5. Conclusions

The inhibition of 5α-R represents a paradigmatic example of how a drug originally designed for urological and dermatological purposes may have repercussions for neurobiology and mental health. The available evidence suggests that while most patients tolerate finasteride and dutasteride adequately, there is a vulnerable subgroup in which a reduction in neurosteroids—particularly allopregnanolone—may contribute to the development of affective and cognitive symptoms.

Epidemiological data are heterogeneous and limited by methodological biases, yet the biological plausibility of this association is well supported. This warrants heightened caution, especially when the indication is cosmetic, and underscores the need to strengthen patient counseling within a shared decision-making framework.

The identification of neuroendocrine and genetic biomarkers, combined with greater methodological rigor in clinical studies, will enable progress toward a personalized medicine approach, allowing clinicians to discriminate between patients who can safely benefit from 5α-R inhibitors and those in whom their use should be avoided.

In summary, the safety of these agents should be re-evaluated from an integrative perspective that encompasses endocrinology, psychiatry, and clinical ethics, with the ultimate goal of maximizing therapeutic benefits without compromising mental health.

## 6. Future Directions

Future research should prioritize the integration of neuroendocrine, genetic, and epigenetic biomarkers to identify individuals at risk of neuropsychiatric adverse effects related to 5α-reductase inhibition. Large-scale prospective studies with standardized psychiatric assessments and direct neurosteroid quantification are needed to overcome current methodological limitations. In addition, the development of isoenzyme-selective inhibitors and strategies that preserve central neurosteroid synthesis while targeting peripheral pathways may offer safer therapeutic options. Finally, pragmatic clinical trials and high-quality pharmacovigilance platforms will be crucial to refine patient selection, optimize dosing strategies, and advance toward a personalized medicine approach.

## Figures and Tables

**Table 1:** Summary of key findings on 5α-reductase isoenzymes, neurosteroid synthesis, psychiatric manifestations, and translational evidence.

Topic	Key Findings	References
Isoenzymes and tissue distribution	Three isoforms have been identified. SRD5A1 is expressed in liver, skin, and brain. SRD5A2 is mainly expressed in prostate and seminal vesicles. SRD5A3 is involved in glycosylation processes and prostate cancer. Together, these isoenzymes participate in peripheral androgen metabolism and neuroendocrine regulation.	[1,2,3,4,5,6,7]
Neurosteroid synthesis	5α-reductase initiates the conversion of progesterone to allopregnanolone. Allopregnanolone is a positive allosteric modulator of GABA-A receptors. It exerts anxiolytic, antidepressant, anticonvulsant, and mood-stabilizing effects. Reduced levels are associated with depression, anxiety, epilepsy, and postpartum depression.	[8,9,10,11,21,22,23]
Psychiatric manifestations of inhibition	Finasteride and dutasteride are effective treatments for BPH and AGA. In a subset of patients, their use has been associated with anxiety, depression, cognitive symptoms, and post-finasteride syndrome. Regulatory agencies (MHRA, EMA, FDA) have issued warnings regarding depression and suicidal ideation.	[12,14,15,17,19]
Clinical and translational studies	Brexanolone, an intravenous allopregnanolone analog, produces rapid improvement in postpartum depression. Zuranolone, an oral analog, is effective in postpartum depression and treatment-resistant major depressive disorder.	[24,25,26,27]
Postmarketing pharmacovigilance studies and healthcare records analyses	Postmarketing pharmacovigilance studies and healthcare database analyses report signals of increased depression, anxiety, and suicidality in finasteride-treated patients. These findings are reported regardless of indication (BPH or AGA). They represent reporting associations and do not establish causality.	[19,28,29,30,31]
Genetic and individual susceptibility	Polymorphisms in SRD5A2 and in GABA-A receptor subunits may explain individual vulnerability. Small-sample studies suggest potential for personalized medicine.	[24,32]

Abbreviations: SRD5A, steroid 5α-reductase; GABA-A, gamma-aminobutyric acid type A receptor; BPH, benign prostatic hyperplasia; AGA, androgenetic alopecia; MHRA, Medicines and Healthcare products Regulatory Agency; EMA, European Medicines Agency; FDA, U.S. Food and Drug Administration.

**Table 2:** Epidemiological studies assessing psychiatric outcomes associated with 5α-reductase inhibitors.

Population/Design	Main Findings	References
FDA pharmacovigilance database (FAERS); postmarketing analysis in AGA	Identified consistent signal of depression, anxiety, suicidal ideation, and suicide in younger men treated with finasteride for alopecia.	[19]
Canadian population-based cohort; >93,000 men with BPH	Slightly increased risk of self-harm and depression during first 18 months of finasteride use; no increased risk of suicide overall.	[28]
French national health insurance database; >200,000 men treated with 5α-R inhibitors (BPH)	Confirmed association between finasteride and increased risk of depression and suicidal ideation in older men with BPH.	[29]
Italian multicenter retrospective cohort; elderly men with BPH	No significant increase in depression or suicidality among finasteride users; study limited to BPH population (not AGA).	[30]
Meta-analysis of observational studies and case reports	Reported heterogeneity in psychiatric outcomes; emphasized need for genetic stratification and better phenotyping.	[32]

Abbreviations: BPH, benign prostatic hyperplasia; AGA, androgenetic alopecia; FDA, U.S. Food and Drug Administration; FAERS, FDA Adverse Event Reporting System.

**Table 3:** Genes and variants associated with neuropsychiatric susceptibility related to 5α-reductase and neurosteroid pathways.

Gene/Variant	Key Findings/Relevance	References
SRD5A3	Involved in glycosylation pathways; mutations associated with congenital glycosylation disorders; role in CNS vulnerability not fully defined.	[6]
SRD5A1	Expressed in liver, skin, brain; polymorphisms may influence allopregnanolone synthesis; preliminary links with stress-related disorders.	[7]
SRD5A2 (A49T polymorphism)	Gain-of-function mutation; increases enzyme activity; reported in urological disease but potential neuropsychiatric implications remain under investigation.	[15]
SRD5A2 (V89L polymorphism)	Reduced enzyme activity; associated with decreased DHT and altered neurosteroid synthesis; linked to vulnerability for depression and anxiety in some cohorts.	[20]
GABRA2 (GABA-A receptor subunit α2)	Variants modulate receptor sensitivity to allopregnanolone; associated with alcohol dependence, anxiety, and mood disorders.	[32]
GABRB3 (GABA-A receptor subunit β3)	Mutations linked to epilepsy, autism spectrum disorder, and mood instability; may interact with neurosteroid modulation.	[42]

Abbreviations: SRD5A, steroid 5α-reductase; DHT, dihydrotestosterone; CNS, central nervous system; GABA-A, gamma-aminobutyric acid type A receptor; GABRA2, GABA-A receptor subunit α2; GABRB3, GABA-A receptor subunit β3.

## Data Availability

The original contributions presented in this study are included in the article. Further inquiries can be directed to the corresponding author.
